# Concrete crack opening forecasting by back propagation neural network and differential equation

**DOI:** 10.1038/s41598-025-11216-2

**Published:** 2025-07-15

**Authors:** Feifei Sun, Zhonghua Xia, Weiqian Feng, Xinhua Zhu, Jinping Xie, Yu Yu, Lvlong Huang, Dong Sheng

**Affiliations:** 1 China Water Resources Beifang Investigation, Design and Research Co., Ltd., 60 Dongting Road, Hexi District, Tianjin, 300222 China; 2 Tianjin Water Resources Research Institute, 60 Youyi Road, Hexi District, Tianjin, 300222 China; 3 Yellow River Conservancy Commission of the Ministry of Water Resources, 11 Jinshui Road, Zhengzhou, 450003 China; 4 Hohai University, 1 Xikang Road, Nanjing, 210024 China; 5 Hunan Water Resources and Hydropower Research Institute, 370 Shaoshan North, Changsha, 410007 China

**Keywords:** Forecast method, Back propagation neural network, Differential equation, Time delay, Northern semiarid region of China, Civil engineering, Scientific data

## Abstract

Concrete crack opening (CCO) is of great importance to hydraulic engineering maintenance. A forecast method is put forward combining back propagation neural network (BPNN) and differential equation (DE) for daily CCO modeling and was applied to Wangqingtuo Reservoir, in the northern semiarid region of China and the contribution of the DE was assessed by using BPNN model as a contrast. First, it is made up of BPNN and DE calibrations: (1) use historical data to calibrate BPNN models and obtain residuals; (2) use the particle swarm optimization to calibrate coefficients of the DE. The periodicity and time delay of air temperature is expressed by the DE well. Second, important results were found by field application: (1) the sole BPNN models can provide reasonable predictions; (2) better prediction can be achieved based on BPNN-DE-2TD by increasing KGE, 12% for JB-1, 37% for JB-3, and 6% for JB-7; (3) it is indicated that the addition of DE can improve the modeling on the role of air temperature under seasonal and linear trend, while BPNN part can express the nonlinear role of water level and precipitation well, confirmed by Fourier amplitude sensitivity test sensitivity and Shapley Additive exPlanations analysis. This study could provide useful insights into further forecasting of CCO under this forecast method in the world.

## Introduction

The structural integrity of hydraulic engineering projects, such as dams, levees, and spillways, is critical for ensuring engineering safety, water resource management, flood control, and energy generation. However, concrete cracks—ranging from superficial surface fissures to deep structural fractures—remain a persistent challenge, significantly compromising the durability and safety of these infrastructures^1–3^. Cracks in hydraulic structures often arise from complex interactions among material properties, environmental stressors, and construction practices^3^.

Early in 1956, it is first categorized^4^ that the factors of concrete displacement are water pressure, temperature, and aging under nonlinear relationship. Thermal gradients during cement hydration, shrinkage due to moisture loss, and uneven mechanical loading can induce tensile stresses exceeding concrete’s capacity, leading to crack initiation and propagation^3,5,6^. Under climate change, temperature’s influence can become more and more severe^7,8^.

Numerical and laboratory studies had been carried out on crack opening^9,10^. A product of axial compression load, slenderness ratio coefficient, eccentric coefficient, and reduction coefficient of the slotted section^9^ is used to express the peak load of specimen in different loading stage. Laboratory study^10^ confirms the impact of basalt fiber on concrete under large eccentric compression, whose deflection and moment can be expressed by differential equation, and whose maximum crack width for large eccentrically compressed columns can be defined by a formula.

Generally, the fracture mechanics and cracking in concrete can summarized. Take concrete filled steel tube columns for example. A few failure modes on concrete filled steel tube columns with huge sections^9^ were summarized: (1) under axial compression, the element columns bend to the outer side and show unstable failure mode, but the connection plates can maintain stable of the slotted column and increase in the bearing capacity; (2) under small eccentric compression, the compression side element column shows the axial compression failure characteristic, and the tensile side element column shows the eccentric compression characteristic; (3) under axial compression or small eccentric compression, all failure locations are below the upper connection plate; (4) under large eccentric compression, all element columns show the eccentric compression failure characteristics, and the failure locations are at the middle height. These failure modes can help partially explain the spatial distribution of cracks in a specific monitoring location to some extent.

Although it is common to use machine learning models on concrete-related studies^11–14^, interestingly, a combination of non-linear finite element model and machine learning techniques can applied to model the load-carrying capacity of concrete-filled steel tubular^15^. A finite-element cohesive zone model was used to consider potential cracks, while a constitutive model was used to account for interface damage and plasticity^16^. Interesting, strain contours with a specified range provided by a finite element model was used to train a deep learning model^12^. A deep learning-based acoustic emission data cluster framework^14^ was developed for evaluating fatigue cracks, which can diagnose overlapping microscopic noise and damage mechanisms across different cases with various crack lengths. As summarized, strong compression, fractures under persistent heavy stresses, temperature variations, structural difficulties, and freeze–thaw cycles can contribute to concrete cracks^17^.

Generally, there are mechanical models^18^, statistical models^7^, and artificial intelligence (AI) models^19^ on concrete crack opening (CCO). Polynomial regression was applied to model the piezometer levels of the Kremasta Dam^20^, while hydrostatic-season-time models were invented for modeling monitoring data^21^.

Since the mechanical models on CCO require plenty of monitoring data and computation power, it is reasonable and feasible to focus more on statistical models and artificial intelligence models. Although there exists conventional statistical models of concrete crack opening^19^, they cannot capture nonlinear features of temperature and other factors^7^ well. Existing models often oversimplify the influence of air temperature^7^, but daily maximum and minimum of air temperature shall be considered^22^. Furthermore, the integration of Back Propagation Neural Network (BPNN) bears good potential^7^ of expressing the nonlinearity, but not time delay of temperature influence. To fill the gap of expressing phase shift, differential equation can be introduced.

The reason why model selection starts from a basic ANN model, BPNN model. And our main goal is to test the feasibility of the DE part whether it can improve the modeling accuracy.

Thus, here a forecast method of BPNN and differential equation (DE) for CCO was put forward to capture both linearity, nonlinearity, and time delay. Our main contributions in this paper are as follows:

- Formulating a BPNN model for daily CCO in training and testing stages;
- Designing a differential equation to express the time delay and nonlinearity on the residual between true observations and model values of the trained BPNN model.

This paper is organized as follows: Section"Forecast method of BPNN-DE"describes the design of the forecasting method based on the Back Propagation Neural Network and Differential Equation (BPNN-DE). Section"Application in the northern semiarid region of China"describes the study region and datasets involved. Section"Results and discussion"presents results and discussion based on the performance of our method. Lastly, Section"Conclusions"concludes the paper, summarizing the results and providing suggestions for further work.

## Forecast method of BPNN-DE

In fact, after we obtained the residual between BPNN model values and measured CCO, we found the residual bears the seasonal and linear trend. As a result, we established the DE part and tested it.

Generally, BPNN as a basic ANN too is to express the nonlinear relationship between CCO and WL and P, while DE is to express the seasonal change and long-term linear change.

### General framework

The CCO forecast problem can be formularized as an AI modeling problem having two composites: one BPNN model and one first order differential equation. First, for one monitoring site in $S=\{1,\dots ,\text{e}\}$ with *e* as the quantity of the forecast sites, the historical data is separated for training and testing a BPNN model, where ${T}_{j,n}=\{1,\dots ,j, 1,\dots ,n\}$ represents the data with *j* as the quantity of time series and *n* as the time. The data includes water level (WL), precipitation, maximum and minimum of air temperature (AT), and CCO values. Second, one DE is designed to model the residual between observed CCO values and values modeled by the BPNN model.

**Fig. 1:**
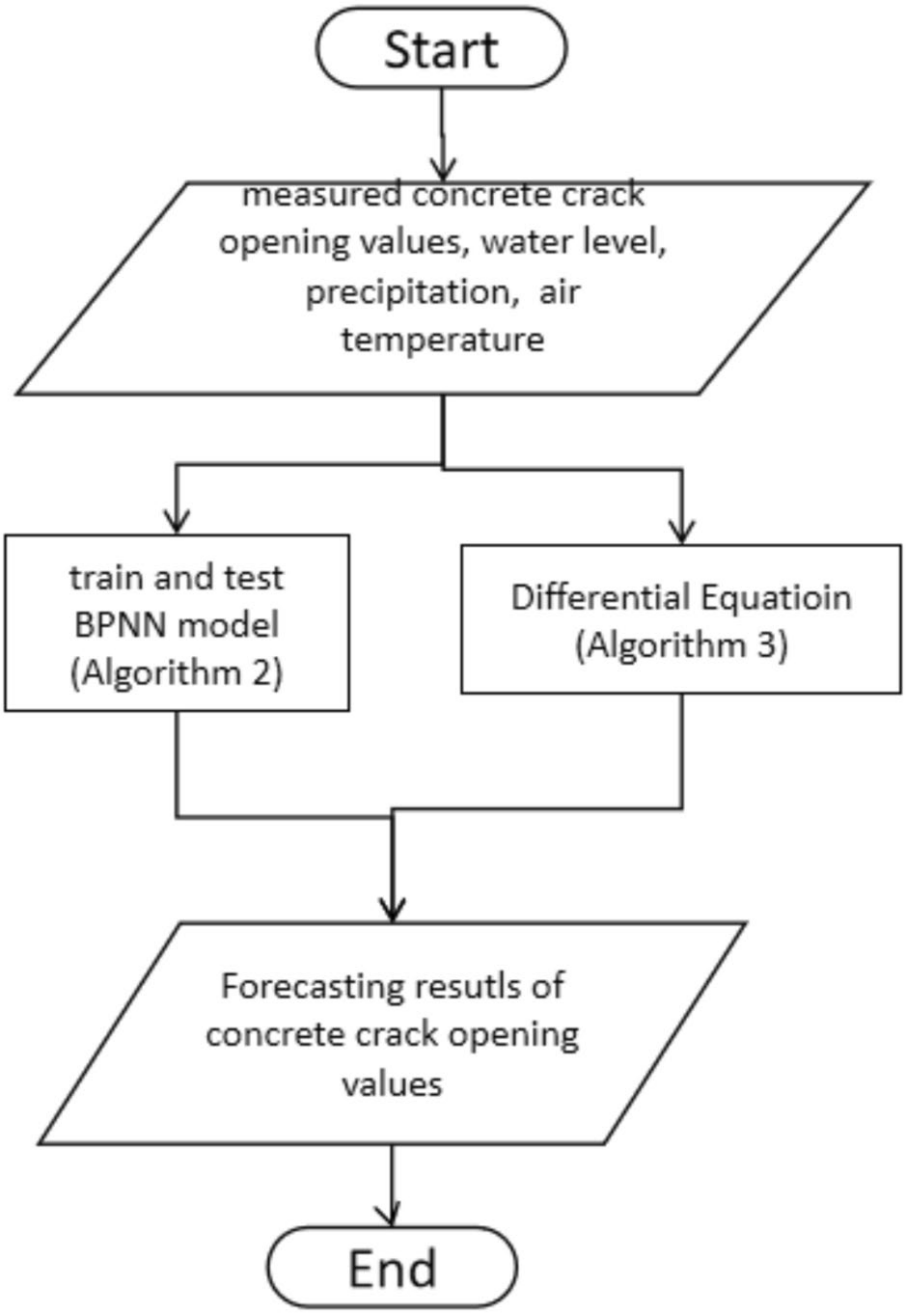
General framework of the forecasting method (Algorithm 1).

The forecast method is shown in Fig. 1 and Algorithm 1. First, use daily data WL, precipitation, maximum and minimum of AT at t-1 to predict CCO at t. Second, BPNN model is trained and tested. Third, a first order differential equation is calibrated on the residual in the training phase, which is the difference between predicted CCO by the trained BPNN model and the measured CCO, correspondingly.

Here, BPNN-DE is described as an optimization problem:

1 $$\text{min }obj=|{f}_{B}({WL}_{m,t-1},{P}_{m,t-1},{MA\_T}_{m,t-1},{MI\_T}_{m,t-1}){-C}_{m,t}|$$

2 $${R}_{t}={C}_{m,t}-{C}_{p,t}={C}_{m,t}-{f}_{B}({WL}_{m,t-1},{P}_{m,t-1},{MA\_T}_{m,t-1},{MI\_T}_{m,t-1})$$

where:

${f}_{B}()$: The trained BPNN model based on measured data of training phase, including WL, precipitation, and maximum and minimum of AT at time t-1; here, the trained BPNN model captures the general nonlinear relationships among these factors, whereas the DE part tries to express the seasonality and nonlinearity of air temperature’s role and others.

- ${WL}_{m,t-1}$: The measured water level at time t-1;
- ${P}_{m,t-1}$: The measured precipitation at time t-1;
- ${MA\_T}_{m,t-1}$: The measured maximum air temperature at time t-1;
- ${MI\_T}_{m,t-1}$: The measured minimum air temperature at time t-1;
- ${C}_{m,t}$: The CCO measured value at time t;
- ${C}_{p,t}$: The CCO predicted value by BPNN at time t;
- ${R}_{t}$: The residual of CCO between predicted by BPNN and measured at time t.

**Figure:**
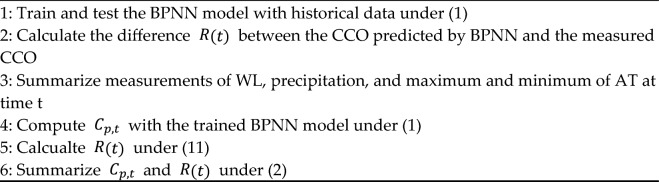
Algorithm 1: Forecast method.

### Back propagation neural network

**Fig. 2:**
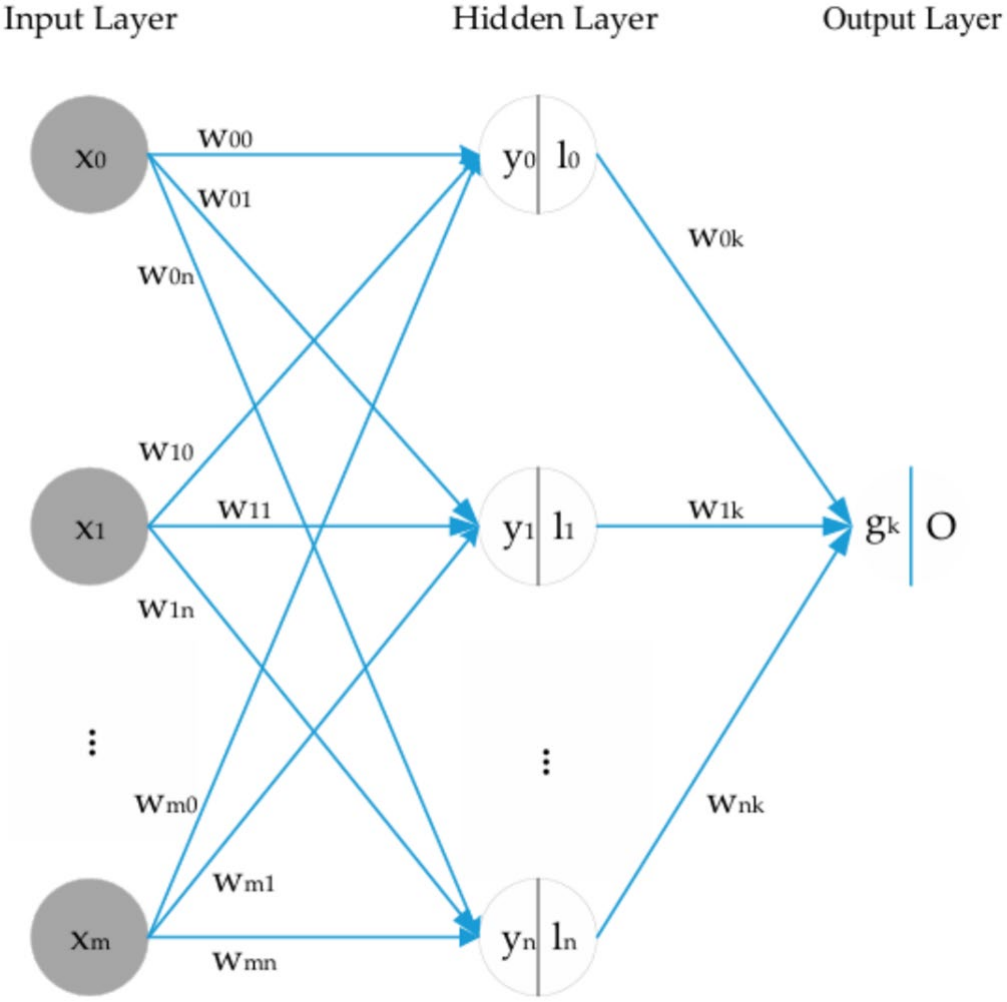
Back propagation neural network structure.

BPNN is a typical multilayer ANN on the basis of error backpropagation^23^. It applies the slope reduction algorithm to minimize error. It is made up of three layers, namely the input layer, hidden layer, and output layer (Fig. 2). While multiple inputs are included in the input layer, one output is included in the output layer. In the hidden layer, multiple neurons bear no direct contact with the outside world, but express the relationship between the input layer and output layer.

A conventional three-layer BPNN is used to establish the prediction model of the daily CCO in this paper. Tan-sigmoid is the transfer function between output and hidden layers, and the nonlinear Levenberg–Marquardt algorithm is the training function of BPNN. The maximum number of iterations is 100. The number of input layer nodes is the same as the number of input variables. The optimal value is determined by continuously adjusting the number of hidden layer neurons in the range of 2 to 13. The original datasets fall into training samples (70%) and testing samples (30%).

After trial and error, two BPNN models were trained and tested with inputs. Both have 4 neuros in the hidden layer.

The mathematical principle of the BPNN model is as follows^23^:

3 $${y}_{i}=\sum_{j=0}^{m}{\omega }_{ij}{x}_{j}+{\beta }_{j}$$

where ${x}_{j}$ is input neuron and $j\in (0, m)$, $m$ is the number of input neurons, ${\omega }_{ij}$ is weight of the *j*th neuron in theinput layer corresponding to the *j*th neuron in the hidden layer, ${\beta }_{j}$ is bias-related weight of hidden neurons, ${y}_{i}$ is input of the hidden layer node (*i* = 0, 1, …, *n*), and *n* the number of neurons in the hidden layer. Tan-sigmoid is the transfer function between the layer output and the hidden layer, and its form is as follows^23^:

4 $${l}_{i}=\frac{1}{1+{e}^{-{y}_{i}}}$$

The output layer is estimated by the following equation^23^:

5 $${g}_{k}=\sum_{i=0}^{n}{\omega }_{ik}{l}_{i}+{\beta }_{k}$$

6 $$O=\text{max}(0, {g}_{k})$$

Among them, ${g}_{k}$ and *O* represent input and output values of the output layer, respectively.

The formulas above are the principles of the feedforward propagation mode of the BPNN model. In the process of cyclic simulation, errors generated by the system are collected and returned to the output value (Algorithm 2). By adjusting the weights and thresholds of neurons, network parameters corresponding to the minimum error are determined to generate an ANN system that can simulate the original problem.

**Figure:**
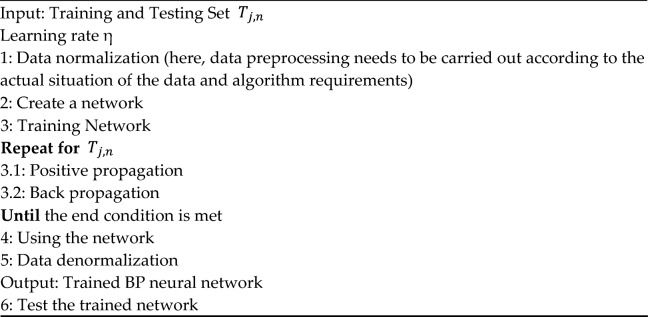
**Algorithm 2: **Back propagation neural network

### Differential equation

A 1^st^ order differential equation is constructed for the residual ${R}_{t}$.

7 $$\frac{dR}{dt}={k}_{1}*\text{cos}\left(\frac{2\pi \left(MOD\left(t,365\right)-TD\right)}{365}+\pi \right)+\frac{{k}_{2}}{365}$$

where:

- ${k}_{1}$: the amplitude coefficient of periodic fluctuation term characterizing the driving strength of crack propagation by the rate of environmental temperature change; probably ${k}_{1}$ is related with material thermal expansion coefficient and daily temperature difference.
- $TD$: the time delay between air temperature and crack opening, which could be a constant, or a function of accumulated temperature on time t; TD can be piecewise function or other complex functions.
- ${k}_{1}*\text{cos}\left(\frac{2\pi \left(MOD\left(t,365\right)-TD\right)}{365}+\pi \right)$: The periodic term, which reflects the impact of seasonal temperature changes on cracks; $MOD\left(t,365\right)-TD$ represents the *TD*th day of each year as the phase reference (which may correspond to the time for heat transferring from air to concrete);
- ${k}_{2}$: The rate constant of linear trend term, which represents the continuous crack propagation caused by material degradation during characterization.
- $\frac{{k}_{2}}{365}$: The linear trend term, which reflects the long-term crack propagation trend caused by material aging/continuous load and is related with time-varying effects such as concrete creep and foundation settlement.

Calculate the integral,

$$R\left(t\right)=\int \left[{k}_{1}*\text{cos}\left(\frac{2\pi \left(MOD\left(t,365\right)-TD\right)}{365}+\pi \right)+\frac{{k}_{2}}{365}\right]dt+C$$

8 $$=\frac{{k}_{1}*365}{2\pi }*\text{sin}\left(\frac{2\pi \left(MOD\left(t,365\right)-TD\right)}{365}+\pi \right)+\frac{{k}_{2}}{365}t+C$$

Assume as $t$ =0, have $R\left(0\right)$,

9 $$R\left(0\right)={R}_{0}=\frac{{k}_{1}*365}{2\pi }*\text{sin}\left(-\frac{2\pi *TD}{365}+\pi \right)+C$$

Under the trigonometric identity $sin\left(\pi -x\right)=\text{sin}(x)$, simplify and have,

10 $$C={R}_{0}-\frac{{k}_{1}*365}{2\pi }*\text{sin}\left(\frac{2\pi *TD}{365}\right)$$

Finally, obtain,

$$R\left(t\right)={R}_{0}+\frac{{k}_{2}}{365}t+\frac{{k}_{1}*365}{2\pi }*\text{sin}\left(\frac{2\pi \left(MOD\left(t,365\right)-TD\right)}{365}+\pi \right)+C$$

11 $$={K}_{1}*\text{sin}\left(\frac{2\pi \left(MOD\left(t,365\right)-TD\right)}{365}+\pi \right)+{K}_{2}t+{C}^{\prime}$$

The detail of differential equation is described in Algorithm 3. First, the calibration of coefficients in (11) is accomplished in the training phase of the BPNN model by using the residual by particle swarm optimization. Second, adopt the coefficients to calculate the residual at time t.

**Figure:**
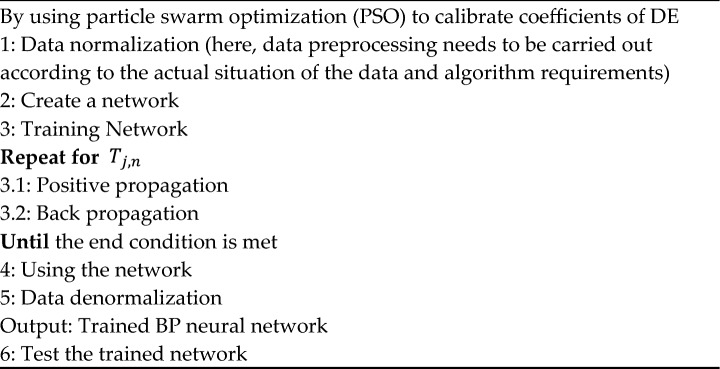
Algorithm 3^25^**:** 1 st order differential equation solved by particle swarm optimization.

## Application in the northern semiarid region of China

### Study region

**Fig. 3:**
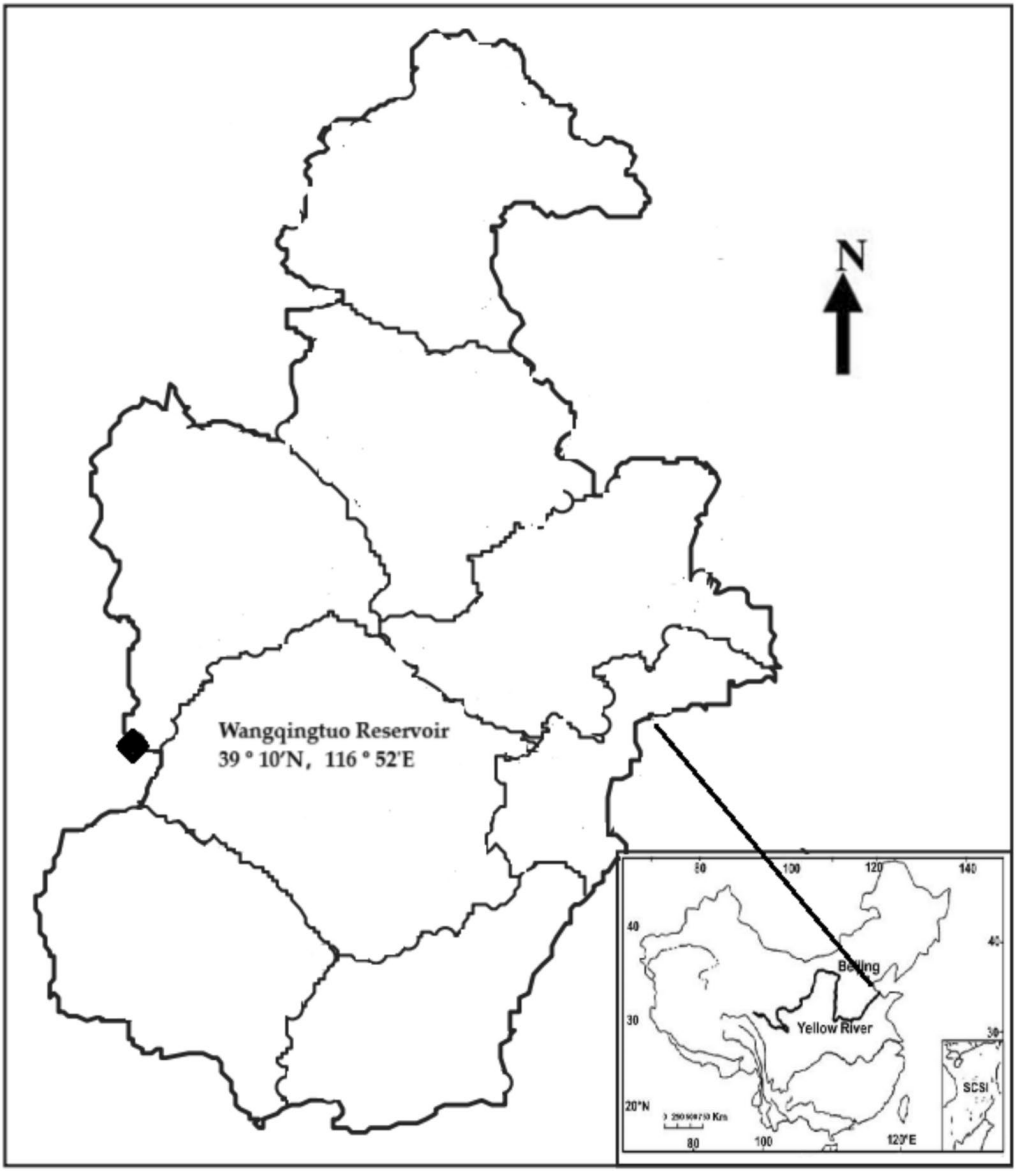
The location of Wangqingtuo reservoir (in Tianjin City) of the northern semiarid region of China.

Wangqingtuo Reservoir^26,27^ is located in the western part of the town of Wangqingtuo in Tianjin, at 39 º 10’N and 116 º 52’E (Fig. 3), and was put into operation in 2019. It is a 24/7 regulating reservoir for the South to North Water Diversion Project and has no watershed cover, with 2000 × 10^4^ m^3^ storage capacity. The main construction contents include reservoir dam, pump station, water return gate, etc. Tianjin city of China is semiarid region, with annual precipitation 534.8 mm.

The most important function of Wangqingtuo Reservoir is: (1) to ensure the smooth switching of water sources from the Yangtze River to the Luan River during maintenance and shutdown of the main canal of the South to North Water Diversion Project; (2) to regulate the unevenness of the incoming water and ensure the stability of urban water supply flow.

### Datasets

**Fig. 4:**
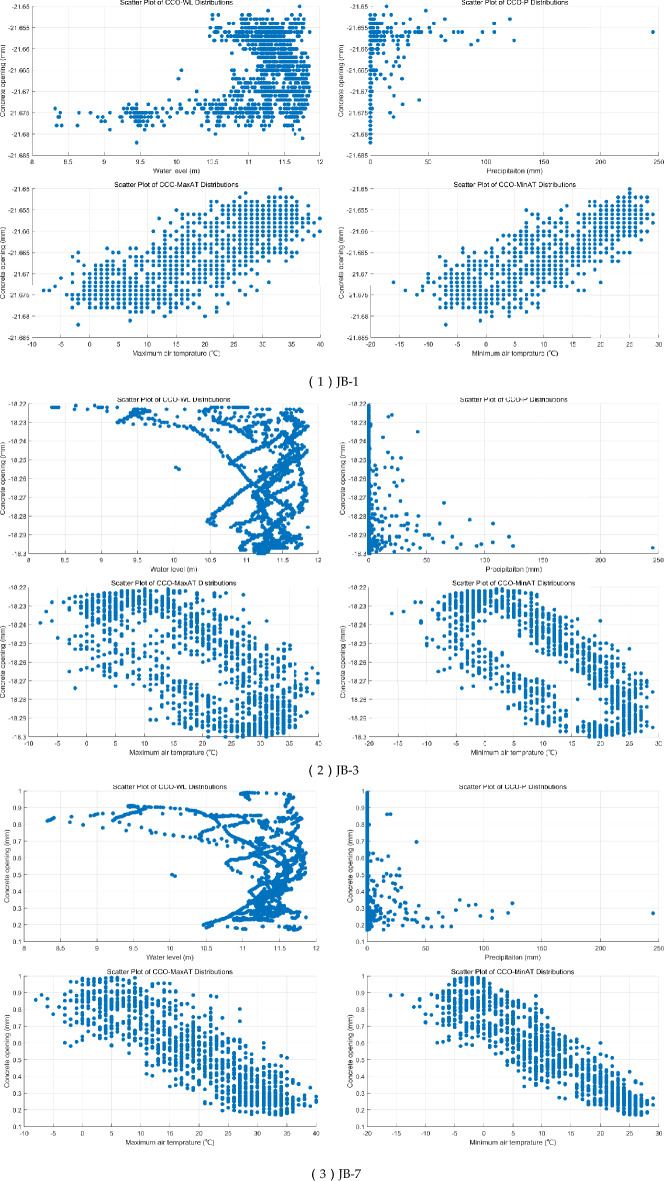

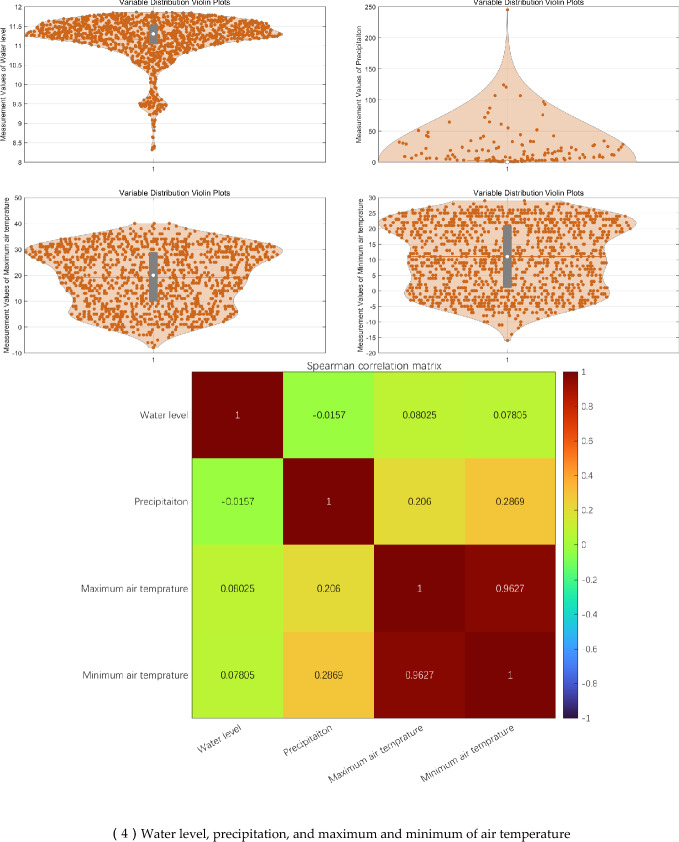
Scatter plots, violin plots, and spearman correlation matrix for statistical data analysis.

Three CCO monitoring sites were involved in this study, including JB-1, JB-3, and JB-7, daily values from 2020–1-3 to 2024–8-10 field daily records of WL, precipitation, and maximum and minimum of AT were applied for modeling. The scatter plots, violin plots, and spearman correlation matrix for statistical data analysis are displayed in Fig. 4. The scatter plots show: (1) JB-3 and JB-7 have similar scatter plots between CCO and WL, a kind of piecewise linear relationships; (2) The scatter plots between CCO and MA_T and MI_T in JB-3 have large hollows inside, while the scatter plot between CCO and MI_T in JB-1 have quite small hollows inside. Generally, the hollows in scatter plots between CCO and MA_T and MI_T seem related to the time delay phenomenon.

Table 1 shows that CCO has higher linear correlation with MA_T and MI_T than WL and P. And CCO of JB-3 has lower linear correlation with MA_T and MI_T than ones of JB-1 and JB-7.

**Table 1:** Spearman matrix for concrete opening measurements.

Item	CCO of JB-1	CCO of JB-3	CCO of JB-7
WL	0.176	0.187	0.173
P	0.036	0.031	0.032
MA_T	0.628	0.441	0.738
MI_T	0.714	0.518	0.817

Table 2 shows the basic statistical characteristics of the whole dataset. The linear relationship between CCO and WL is low, which cannot deny that there exists strong nonlinear relationship between CCO and WL.

**Table 2:** Basic statistical characteristics of the dataset.

Item	WL	P	MA_T	MI_T	CCO of JB-1	CCO of JB-3	CCO of JB-7
Minimum	8.32	0.00	−8.00	−16.00	−21.68	−18.30	0.16
Maximum	11.86	245.0	40.00	29.00	−21.65	−18.22	0.99
Mean	11.17	2.14	19.26	11.05	−21.66	−18.26	0.52
Standard deviation	0.62	11.42	11.34	10.70	0.01	0.03	0.23
Skewness	−1.87	10.33	−0.28	−0.16	−0.04	0.0016	0.21

The measurement is complete and automatic. Data cleaning, missing value handing, normalization, and seasonality adjustment are not involved.

This study adopted the measured values of WL, precipitation, and maximum and minimum of AT from Meteorological department of Tianjin with the sole goal of evaluating this forecast method.

### Study design under the method

First, we use the historical 2,020,103–20,230,810 datasets to train and test one BPNN model (see Algorithm 2).

Second, based on the difference between measured CCO and prediction CCO by the BPNN model during 2,020,103–20,230,810, PSO (see Algorithm 3) is used to calibrate the coefficients of the DE part.

Third, use the measured values during 20,230,811–20,240,810 to make BPNN predictions of CCO and use the calibrated DE to obtain DE predictions of CCO. Finally summarize these two parts.

Fourth, modeling is evaluated by the R^2^, maximum error, minimum error, average absolute error, the ratio with < 20% error, and Kling Gupta efficiency (KGE) in the training stage, testing stage, and forecasting stage. As R^2^ is larger than 0.8, the modeling performs well. The closer the KGE goes to 1, the better the modeling is. The Fourier amplitude sensitivity test (FAST) sensitivity analysis, Shapley Additive exPlanations (SHAP) analysis, and Taylor analysis were carried out. We also adopted Variance Accounted For (VAF), Entropy, and mutual information (MI), and Total Information Criterion (TIC) for analysis. The SPSS modeler and MATLAB are applied to carry out the modeling and analysis.

## Results and discussion

### Training, testing, and forecasting

As can be seen from Table 3, the only BPNN models do not perform well with R^2^ not that large. According *Standard for hydrological information and hydrological forecasting (GB/T 22,482–2008)*^28^, as the ratio with < 20% absolute error is larger than 85%, the forecasting is considered first-class. In a word, our forecasting in JB-1 and JB-3 by the BPNN model (see Table 4) meets the first-class criterion.

**Table 3:** Performance of only BPNN model without the DE analytic solution.

Item	JB-1 with mean −21.67			JB-3 with mean −18.23			JB-7 with mean 0.95		
	Train	Test	Forecast	Train	Test	Forecast	Train	Test	Forecast
The ratio < 20% error	100%	99.5%	98.1%	100%	98.6%	97.5%	73.7%	75.9%	72.1%
Average absolute error (% mm)	0.013%	0.014%	0.014%	0.07%	0.075%	0.079%	14.99%	14.57%	14.68%
Minimum error (% mm)	0%	0.001%	0.003%	0%	0.02%	0.06%	0%	0%	0.02%
Maximum error (% mm)	0.060%	0.059%	0.073%	0.219%	0.354%	0.329%	85.45%	85.45%	84.86%
R^2^	0.78	0.772	0.759	0.619	0.604	0.591	0.856	0.887	0.853
Kling Gupta efficiency	0.838	0.834	0.816	0.700	0.695	0.698	0.897	0.892	0.885

**Table 4:** Performance of forecasting method through BPNN-DE.

Item	JB-1 with mean −21.67			JB-3 with mean −18.23			JB-7 with mean 0.95		
Model	BPNN	BPNN-DE-1TD	BPNN-DE-2TD	BPNN	BPNN-DE-1TD	BPNN-DE-2TD	BPNN	BPNN-DE-1TD	BPNN-DE-2TD
${K}_{1}$	**/**	0.005	0.005	**/**	−0.0200	−0.0200	**/**	−0.11	−0.11
${K}_{2}$	**/**	0.001	0.001	**/**	0.0001	0.0001	**/**	0.000005	0.000005
${C}{\prime}$	**/**	−0.0001	−0.0001	**/**	−0.0008	−0.0008	**/**	−0.0085	−0.0085
TD1	**/**	26	26	**/**	23	23	**/**	16.5	16.5
TD2	**/**	26	17	**/**	23	10	**/**	16.5	11
The ratio < 20% error	100%	100%	100%	100%	100%	100%	73.7%	77.8%	79.2%
Average absolute error (% mm)	0.013% **(0.0028)**	0.013% **(0.0027)**	0.012% **(0.0027)**	0.07% **(0.013)**	0.036% **(0.007)**	0.034% **(0.006)**	14.99% **(0.068)**	13.10% **(0.060)**	12.62% **(0.058)**
Minimum error (% mm)	0% **(0)**	0% **(0)**	0% **(0)**	0% **(0)**	0% **(0)**	0% **(0)**	0% **(0)**	0% **(0)**	0% **(0)**
Maximum error (% mm)	0.060% **(0.013)**	0.063% **(0.014)**	0.063% **(0.014)**	0.219% **(0.040)**	0.135% **(0.025)**	0.127% **(0.023)**	85.45% **(0.278)**	74.32% **(0.264)**	66.86% **(0.236)**
R^2^	0.780	0.867	0.877	0.619	0.896	0.916	0.856	0.892	0.903
KGE	0.838	0.926	0.936	0.700	0.912	0.956	0.897	0.914	0.951
VAF	0.778	0.864	0.870	0.619	0.895	0.913	0.858	0.893	0.901
Entropy	4.413	10.355	10.356	6.140	10.716	10.716	8.832	10.606	10.606
MI	1.24	2.48	2.46	2.31	3.58	3.59	4.88	4.98	4.99
TIC	132.85	131.76	131.73	138.83	137.34	136.70	137.55	137.85	138.46

With the PSO algorithm, the coefficients were calibrated for BPNN-DE models (see Table 4). Based on vertex and valley points of air temperatures,, here adopt the February 11^st^ and July 24^th^ as the split points to separate the time.

Thus, under BPNN-DE models, there are BPNN-DE-1TD and BPNN-DE-2TD. For BPNN-DE-1TD, only TD1 is applied for all residual in Eq. (11). For BPNN-DE-2TD, as t falls between February 11^st^ and July 24^th^, TD1 is applied and as t is outside this period, TD2 is applied.

As can be seen from Table 4, both BPNN-DE models with one TD and two TDs perform better than BPNN models, respectively especially by R^2^ and KGE. The KGE increases from BPNN to BPNN-DE-1 TD, 11% for JB-1, 30% for JB-3, 2% for JB-7, while it increases from BPNN to BPNN-DE-2 TD, 12% for JB-1, 37% for JB-3, 6% for JB-7. In general, the addition of DE part can increase model performance, and models with two TDs can add more accuracy than models with one TD, which confirms the time delay influenced by air temperature^7^. VAF, Entropy, MI and TIC confirm that the addition of TD can improve the modeling performance.

**Fig. 5:**
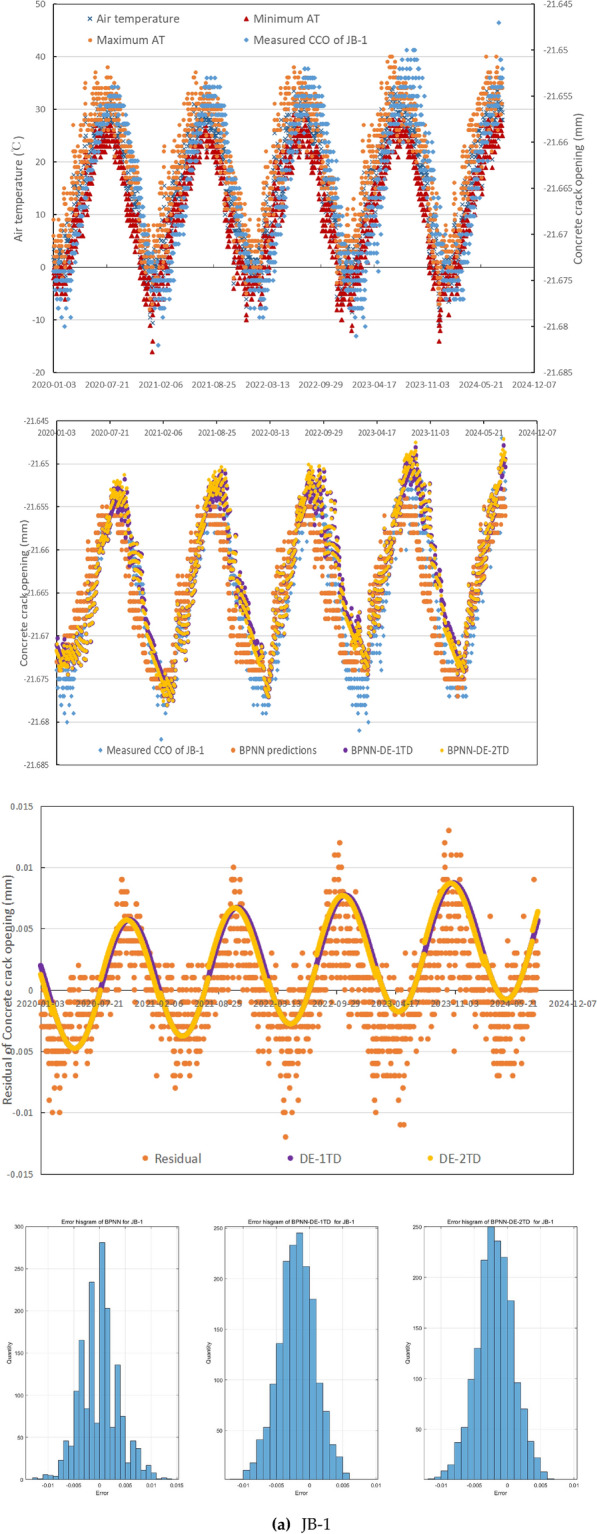

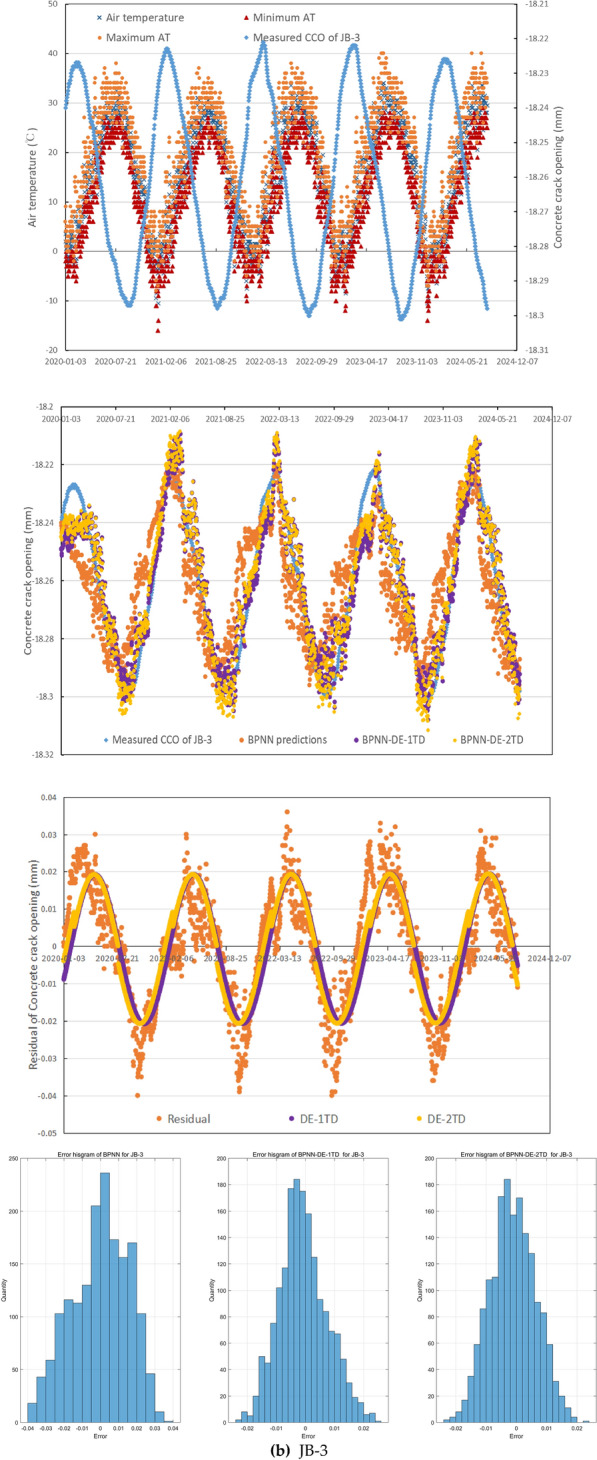

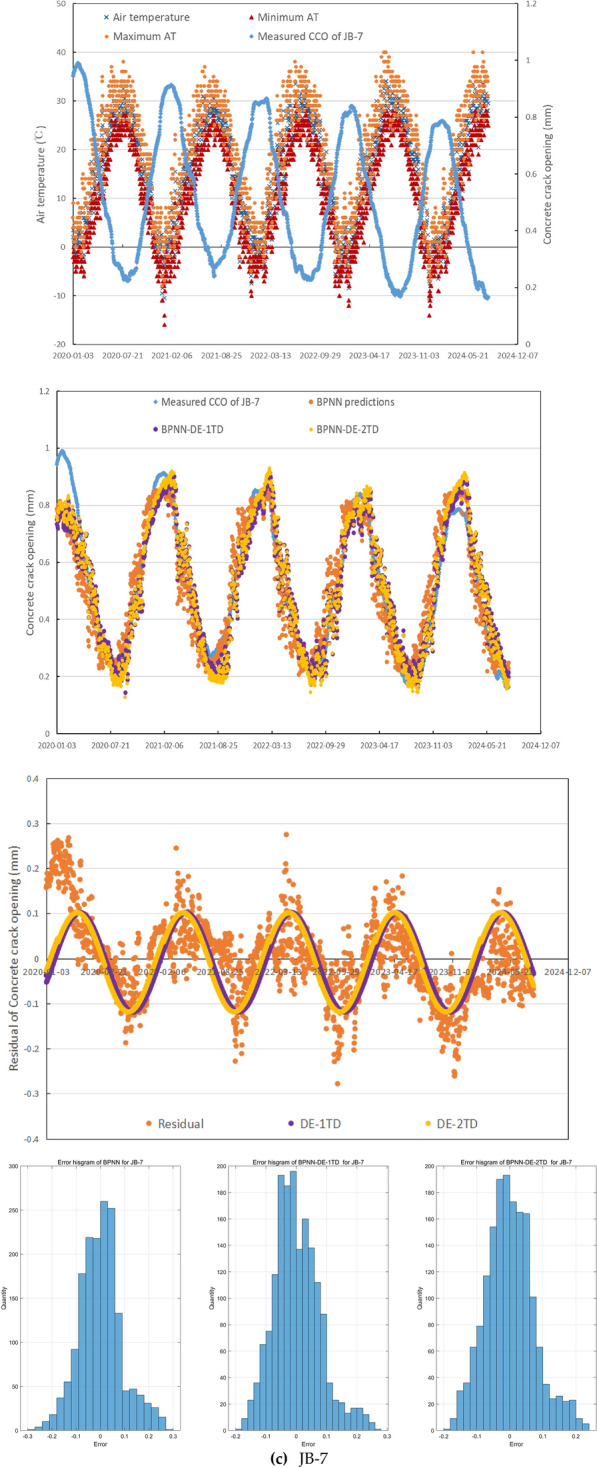
The performance of the forecasting method.

In general, these three CCO monitoring sites emerge different relationship features between measured CCO and mean, maximum, and minimum of AT (see Fig. 5), which can reflect the universality and generality of the mechanism of crack occurrence to some extent in the northern semiarid region of China, besides small daily precipitation, small daily WL change. During 2,020,103–20,230,810, maximum, minimum, and mean of daily WL is 11.86m, 8.32m, and 11.16m. For 1960–2024, mean of annual precipitation is 534.8mm.

In JB-1, while WL and precipitation have poor R^2^ with CCO, less than 0.18, mean, maximum, and minimum of AT have higher R^2^, 0.63, 0.71, and 0.68 respectively. While AT has quite positive relationship with CCO, both AT and CCO change with large random fluctuation with time delay and not good continuity, correspondingly, see Fig. 5(a). It is obvious that the time delay is different in AT rising period and AT falling period based on maximum AT and CCO. As maximum AT rises, the measured CCO is scatted to the right of the scatted maximum AT around 20 days. As maximum AT falls, the measured CCO and maximum AT are scattered quite closer. CCO in JB-1 during 2,020,103–20,230,810 has mean value −21.67 mm with a bit increasing trend. This error histograms for BPNN, BPNN-DE-1TD, and BPNN-DE-2TD in Fig. 5(a) show under the addition of DE and TD: (1) the error range becomes smaller; (2) more errors get closer to zeroes.

In JB-3, while WL and precipitation have poor R^2^ with CCO, less than 0.19, mean, maximum, and minimum of AT have a little higher R^2^, 0.44, 0.52, and 0.49 respectively. While AT has quite negative relationship with CCO, CCO changes with small random fluctuation with time delay and good continuity as AT changes seasonally with large random fluctuation, see Fig. 5(b). It is obvious that the time delay is significant between AT extremes and CCO extremes. CCO in JB-3 during 2,020,103–20,230,810 has mean value −18.23 mm with no obvious trend. This error histograms for BPNN, BPNN-DE-1TD, and BPNN-DE-2TD in Fig. 5(b) show under the addition of DE and TD: (1) the error range becomes smaller; (2) more errors get closer to zeroes.

In JB-7, while WL and precipitation have poor R^2^ with CCO, less than 0.17, mean, maximum, and minimum of AT have higher R^2^, 0.74, 0.82, and 0.79 respectively. While AT has quite negative relationship with CCO, CCO changes with small random fluctuation with time delay and good continuity as AT changes seasonally with large random fluctuation, see Fig. 5(c). It is obvious that the time delay is significant between AT extremes and CCO extremes. CCO in JB-7 during 2,020,103–20,230,810 has mean value −0.95 mm with significant decreasing trend. This error histograms for BPNN, BPNN-DE-1TD, and BPNN-DE-2TD in Fig. 5(c) show under the addition of DE and TD: (1) the error range becomes smaller; (2) more errors get closer to zeroes.

As can be seen from Fig. 5(a), BPNN predictions in JB-1 generally capture the trend, but they have obvious time delay and point-scattered issues, and they cannot express the peaks and bottoms of CCO well. The addition of DE through BPNN-DE-1TD and BPNN-DE-2TD models do help decrease the time delay issue and reduce the point-scattered issue. More, both models with DE indeed improve the accuracy in peaks of predictions, but not those values of bottoms.

As can be seen from Fig. 5(b), BPNN predictions in JB-3 generally capture the trend, but they have obvious time delay and point-scattered issues, and they cannot express the bottoms of CCO well. The addition of DE through BPNN-DE-1TD and BPNN-DE-2TD models do help decrease the time delay issue and reduce the point-scattered issue. While both models with DE indeed improve the accuracy in peaks and bottoms of predictions, the DE models have prediction inefficiency around peaks of 2020, 2022, and 2023.

As can be seen from Fig. 5(c), BPNN predictions in JB-7 generally capture the trend and are good in bottoms and not good in peaks, but they have obvious time delay and point-scattered issues. The addition of DE through BPNN-DE-1TD and BPNN-DE-2TD models do help decrease the time delay issue and reduce the point-scattered issue. While both models with DE indeed improve the accuracy in peaks and bottoms of predictions, the DE models have prediction inefficiency around peaks of 2020, 2021, and 2024 and around bottoms of 2021.

To sum up, the addition of DE to BPNN does increase the accuracy by reducing the point-scattered issue and the addition of TD can fix the time delay to some extent. BPNN-DE-2TD can increase predictions in peaks and bottoms of CCO to some extent.

### Why the BPNN-DE-2TD performs good or not?

To investigate why BPNN-DE-2TD performs good, ahead of all, it is necessary to sort out the factors of concrete crack opening. Generally, there are a few factors contributing to the concrete crack opening, including water pressure, precipitation, temperature, and other material properties and mechanical characteristics. In the operation period of hydraulic engineering, WL, precipitation, AT, and aging are the main factors^4^. While mechanical models has their limits due to require plenty of monitoring data and computation power, statistical and AI models have great application potential^7^.

Second, the combination of BPNN and DE in this paper has shown great both theory and application meaning for modeling concrete crack opening (see Fig. 5). On one hand, BPNN demonstrates powerful nonlinear modeling capabilities on small and medium-sized datasets through hierarchical nonlinear transformations and error feedback mechanisms, but the Sigmoid function is prone to gradient exponential decay in deep networks^24,29^. On the other hand, DE provides a complete modeling language from deterministic to stochastic, from continuous to discrete^30^. While the sine/cosine function is good at expressing the seasonal variations, the TDs can reflect the time delay of the influence of air temperature very well (see Fig. 5).

Third, the research target Wangqingtuo Reservoir is unique in some ways. First, it has no watershed, which means in two ways: (1) its WL is not influenced by precipitation generating runoff from a watershed; (2) its WL is predictable by calculating the water volume in or out under one-day-ahead water-use planning. More it is quite shallow, with design water level 11.9 m, the dead water level 6.47 m, the bottom elevation 4.2 m, and maximum water depth 7.7 m. As a result, water pressure plays little role on CCO. Second, without watershed, it only absorbs the precipitation with its reservoir area 3.92 km^2^. Thus, precipitation plays little role on CCO. To sum up, the air temperature and other material or mechanical factors are the main contributor of CCO in Wangqingtuo Reservoir.

Forth, although BPNN-DE-2TD performs good to some extent, it has some shorts in predicting some peaks or bottoms. First, the DE analytic solution do not use the AT time series just by using Mod(t, 365), which can be improved probably by a function of the accumulated temperature. Second, the TD is segmented constant term, which can be improved by introducing a function of the AT. Third, since Wangqingtuo Reservoir is mainly influenced by AT, DE on WL and Precipitation for other hydraulic engineering can be a good research topic.

### The physics behind BPNN-DE-2TD

**Fig. 6:**
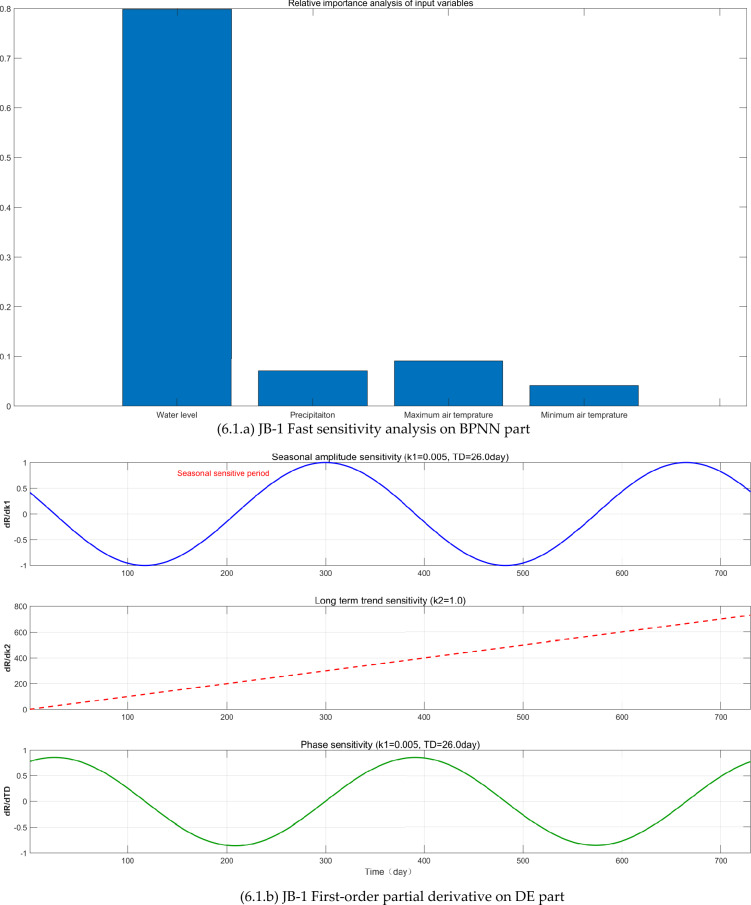

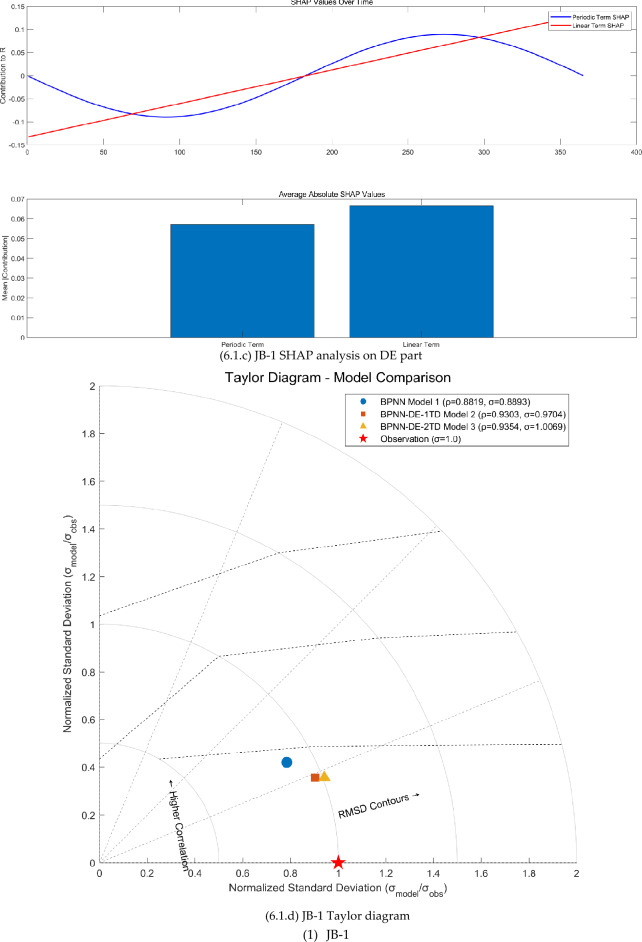

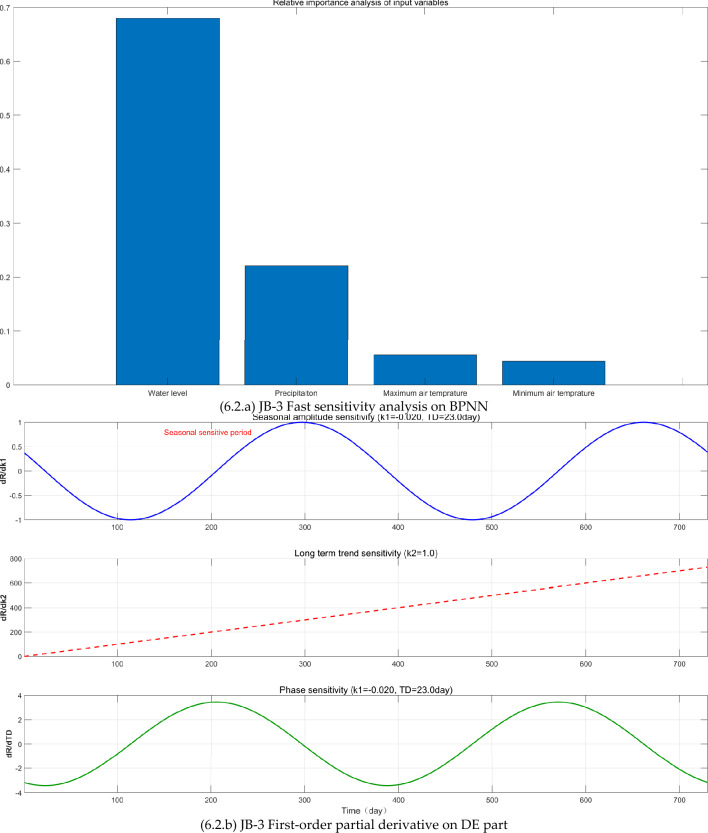

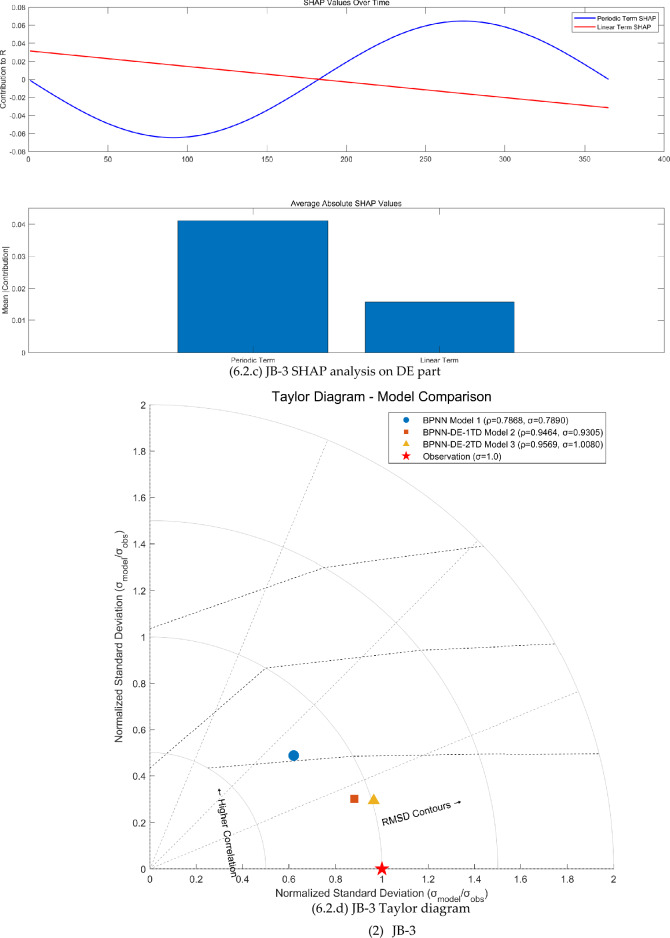

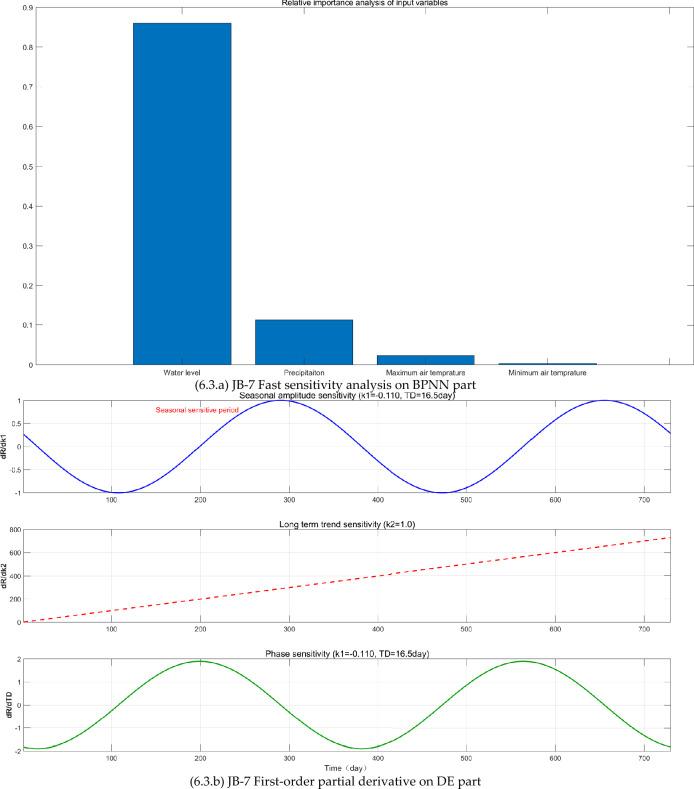

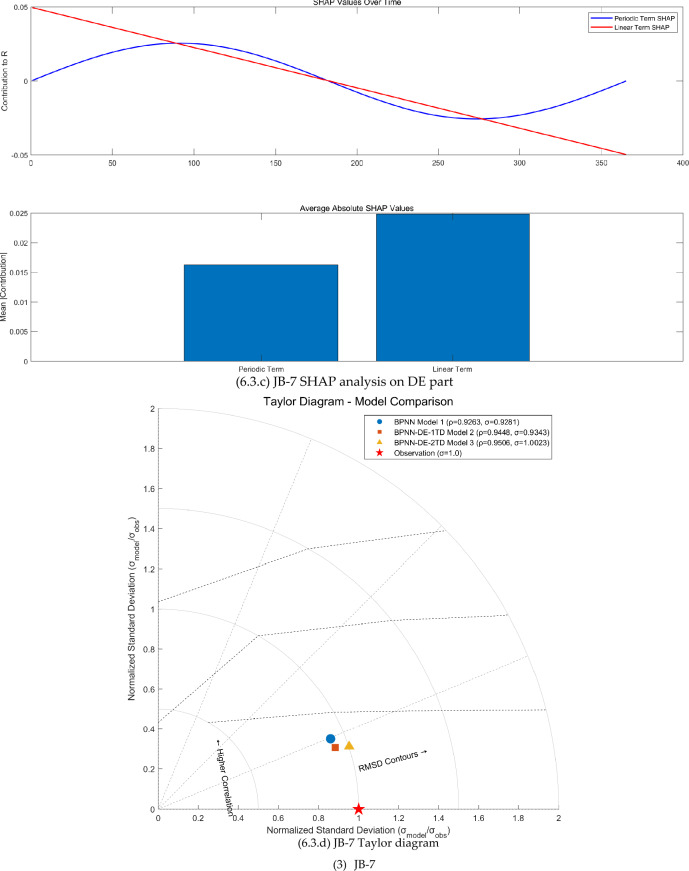
The FAST sensitivity analysis, SHAP analysis, and Taylor analysis.

The FAST sensitivity analysis, SHAP analysis, and Taylor analysis were carried out (see Fig. 6) to facilitate to understand the physics behind the BPNN-DE-2TD model.

First, BPNN expresses the nonlinear relationship between CCO and WL, P, MA_T, and MI_T, proved by the relative importance values of FAST sensitivity analysis (see Fig 6.a, 6.a, and 6.a). WL and P are the most important factors. WL’s relative importance (RI) values in JB-1, JB-3, and JB-7 are 0.79, 0.68, and 0.87, respectively, while P’s RI values are 0.07, 0.23, and 0.12. JB-1 and JB-7 have the elevation 9 m while JB-3 is −1m. JB-1 and JB-3 are 42 m away from the Wangqingtuo dam’s external foot, while JB-1 is right at the external foot, 0m.

Although the monitoring sites are limited, here probably it is inferenced from the FAST sensitivity analysis on BPNN part that: (1) P can increase the RI value by increasing the underground water level. JB-3’s elevation is −1m, which can be affected by the underground water level. Consequently, JB-3 has the largest RI value 0.23. (2) the increase of distance to the water in the Wangqingtuo reservoir can reduce the RI value. As the distance increases from 0 m of JB-7 to 42 m of JB-1, the RI decreases from 0.87 of JB-7 to 0.79 of JB-1. (3) as the elevation goes low from 9 m of JB-1 to −1m of JB-3, the WL’s RI goes low from 0.79 to 0.68.

Second, DE expresses the seasonal variation, linear trend, and time delay, confirmed by first-order partial derivative (see Fig 6.b, 6.b, and 6.b) and SHAP analysis (see Fig 6.c, 6.c, and 6.c). First-order partial derivative on DE part shows the seasonal amplitude sensitivity in the form of shifted sine function, long term trend sensitivity in the form of linear equation, and phase sensitivity in the form of shifted cosine function. Average absolute SHAP values of periodic term and linear term for JB-1, JB-3, and JB-7 are 0.057 and 0.068, 0.039 and 0.017, 0.016 and 0.025. The ranges of JB-1, JB-3, and JB-7 are 0.03mm, 0.07mm, and 0.83mm. Based on average absolute SHAP values and ranges, it seems that the small the range is, the large the average absolute SHAP values of DE part are. As the range becomes larger, periodic term and linear term of the DE part make smaller contribution.

Third, the TD does increase the modeling accuracy, proved by Taylor diagrams (see Fig. 6.1.d, Fig. 6.2.d, and Fig. 6.3.d). For JB-1, JB-3, and JB-7, BPNN-DE-2TD model performs better than BPNN-DE-1TD, which is better than BPNN model. It is worth noting that standard deviation of BPNN-DE-2TD goes to 1 for all monitoring sites. Two TDs offer more accuracy by assuming that there exits different TDs in heating process and cooling process.

## Conclusions

It is important to model concrete crack opening. Inspired by related researchers, a forecast method by combining the BPNN and DE for measured CCO is put forward. First, historical data is used to calibrate BPNN models and obtain residuals. Second, the PSO is used to calibrate the DE for the residuals. Third, summarize the BPNN and DE parts.

This forecasting method was applied in the Wangqingtuo Reservoir’s 2020–2024 daily datasets. The application offers important results: (1) the sole BPNN models can provide reasonable predictions; (2) the method through BPNN-DE-2TD can achieve better prediction of great significance by increasing KGE, 12% for JB-1, 37% for JB-3, and 6% for JB-7; (3) BPNN-DE-2TD can model the influence of air temperature well, not water level, and precipitation. Generally, DE can express the role of air temperature under seasonal and linear trend, while BPNN part can express the nonlinear role of water level and precipitation, confirmed by FAST sensitivity and SHAP analysis.

Admittedly, the forecast method is new, and has some limitations, like not considering time-vary TD or time-varying WL. For example, admittedly, WL changes influence the CCO propagation. In our opinion, the time-varying WL changes could possibly be integrated into the DE part in the form of a new subitem.

## Data Availability

The data related with this study will be available based on reasonable request through corre-sponding author.

## Abbreviations

AT: Air temperature
BPNN: Back propagation neural network
BPNN-DE-2TD: Back propagation neural network-differential equation with two TDs
BPNN-DE-1TD: Back propagation neural network-differential equation with one TD
C: Concrete crack opening value
CCO: Concrete crack opening
DE: Differential equation
${f}_{B}$: The trained BPNN model
FAST: Fourier amplitude sensitivity test
MA_T: Maximum air temperature
MI_T: Minimum air temperature
KGE: Kling Gupta efficiency
P: Precipitation
R: Residual
RI: Relative importance
SHAP: Shapley additive exPlanations
TD: Time dely
WL: Water level
